# Metabolic analysis of the regulatory mechanism of sugars on secondary flowering in *Magnolia*

**DOI:** 10.1186/s12860-022-00458-x

**Published:** 2022-12-14

**Authors:** Lingjuan Xuan, Qianqian Wang, Zhigao Liu, Bin Xu, Shaoyu Cheng, Yingjia Zhang, Danying Lu, Bin Dong, Dongmei Zhang, Lang Zhang, Jingjing Ma, Yamei Shen

**Affiliations:** 1 School of Landscape and Architecture, Zhejiang A&F University, Zhejiang, 311300 Hangzhou China; 2 Shanghai Academy of Landscape Architecture Science and Planning, Shanghai, 200232 China

**Keywords:** *Magnolia*, Flower bud differentiation, *MlTPS* genes, Sucrose and trehalose spraying, Metabolite analysis

## Abstract

**Background:**

*Magnolia*, a traditional and important ornamental plant in urban greening, has been cultivated for about 2000 years in China for its elegant flower shape and gorgeous flower color. Most varieties of *Magnolia* bloom once a year in spring, whereas a few others, such as *Magnolia liliiflora* Desr. ‘Hongyuanbao’, also bloom for the second time in summer or early autumn. Such a twice flowering trait is desirable for its high ornamental value, while its underlying mechanism remains unclear.

**Methods:**

Paraffin section was used to show the flowering time and phenotypic changes of *M. liliiflora* ‘Hongyuanbao’ during the twice flowering periods from March 28 to August 25, 2018. Gas chromatography-mass spectrometry (GC-MS) was then performed to explore the chemical metabolites through the twice flower bud differentiation process in ‘Hongyuanbao’, and the metabolites were screened and identified by orthogonal projection to latent structures discriminant analysis (OPLS-DA). Kyoto Encyclopedia of Genes and Genomes pathway enrichment analysis (KEGG) was used to reveal the relationship between the sugar metabolites and twice-flowering characteristic. To further investigate the potential role of sucrose and trehalose on flowering regulation of ‘Hongyuanbao’, the plants once finished the spring flowering were regularly sprayed with sucrose and trehalose solutions at 30 mM, 60 mM, and 90 mM concentrations from April 22, 2019. The flower bud differentiation processes of sprayed plants were observed and the expression patterns of the genes involved in sucrose and trehalose metabolic pathways were studied by quantitative reverse transcription PCR (qRT-PCR).

**Results:**

It showed that ‘Hongyuanbao’ could complete flower bud differentiation twice in a year and flowered in both spring and summer. The metabolites of flower bud differentiation had a significant variation between the first and second flower buds. Compared to the first flower bud differentiation process, the metabolites in the sucrose and trehalose metabolic pathways were significantly up-regulated during the second flower bud differentiation process. Besides that, the expression levels of a number of trehalose-6-phosphate synthase (*TPS*) genes including *MlTPS1*, *MlTPS5*, *MlTPS6*, *MlTPS7* and *MlTPS9* were substantially increased in the second flower differentiation process compared with the first process. Exogenous treatments indicated that compared to the control plants (sprayed with water, CK), all three concentrations of trehalose could accelerate flowering and the effect of 60 mM concentration was the most significant. For the sucrose foliar spray, only the 60 mM concentration accelerated flowering compared with CK. It suggested that different concentration of trehalose and sucrose might have different effects. Expression analysis showed that sucrose treatment increased the transcription levels of *MlTPS5* and *MlTPS6*, whereas trehalose treatment increased *MlTPS1*, showing that different *MlTPS* genes took part in sucrose and trehalose metabolic pathways respectively. The expression levels of a number of flowering-related genes, such as *MlFT, MlLFY*, and *MlSPL* were also increased in response to the sprays of sucrose and trehalose.

**Conclusions:**

We provide a novel insight into the effect of sucrose and trehalose on the flowering process in *Magnolia*. Under the different sugar contents treatments, the time of flower bud differentiation of *Magnolia* was advanced. Induced and accelerated flowering in response to sucrose and trehalose foliar spray, coupled with elevated expression of trehalose regulatory and response genes, suggests that secondary flower bud formation is a promoted by altered endogenous sucrose and trehalose levels. Those results give a new understanding of sucrose and trehalose on twice-flowering in *Magnolia* and provide a preliminary speculation for inducing and accelerating the flowering process in *Magnolia.*

**Supplementary Information:**

The online version contains supplementary material available at 10.1186/s12860-022-00458-x.

## Background

Flowering is an prominent feature and essential part of reproductive process in flowering plants, which constitute the largest and most diverse group of the plant kingdom [1, 2]. The flowering time is critical in the life cycle of a plant to ensure maximum reproductive success [3–5]. During the process of cultivation and breeding, ornamental plants bear flowers with rich colors and changeable flowering time, and longer flowering periods are generally favored [2]. Among the flowering plants, most bloom once a year and these plants are called as once flowering plants [6]. While some plants can bloom again within a year as twice flowering plants or continue to bloom under favorable conditions as continuous flowering plants [6].

In recent years, there have been a number of in-depth studies on the molecular mechanisms of plant flowering, especially in model plant *Arabidopsis thaliana*. It has found that six major pathways including photoperiod, vernalization, thermosensory, gibberellin, autonomous, and aging regulate flower development process [7, 8]. Many genes have been found to integrate the signals received via these pathways, such as *FT* (*FLOWER LOCUS T*), *AP1* (*APETALA1*) and *LFY* (*LEAFY*) [9]. In *A. thaliana*, *FT* serves as the florigen molecule that transmits this integrated flowering signal to the shoot apical meristem to trigger flowering [10]. *AP1* and *LFY* integrate the signals of multiple floral induction pathways and activate floral organ development [11]. These integrative genes are highly conserved in plants, including apple [12], longan [13], and poplar [14].

In addition, sugars and metabolic pathways are known to influence flowering, and there are changes in metabolites associated with flowering process [15, 16]. Sugars were found to act as signaling molecules interacting with flowering-control pathways, as exemplified by the transcription factor *INDETERMINATE DOMAIN8* (*AtIDD8*) which controls flowering by regulating sucrose transport and metabolism in *A. thaliana* [17]. Additionally, trehalose is involved in many important metabolic and developmental processes regulation in flowering plants [15, 18–20]. Trehalose-6-phosphate (T6P) is synthesized from UDP-glucose and glucose-6-phosphate by T6P synthase (*TPS*) and then converted to trehalose by T6P phosphatase [21, 22]. Since T6P exists in plants in trace amounts, it is thought to act as a signaling molecule for the regulation of sugars [23]. In many plants, the content of T6P is closely related to that of endogenous sucrose [15]. This significant correlation suggests that T6P may be a signal of sucrose availability and a negative feedback regulator of sucrose accumulation [18]. In studies of *A. thaliana*, it shows that the T6P pathway can directly regulate flowering at two sites: First, *TPS1* activity is required for the induction of *FT,* which has a central role in flowering time control by integrating signals from the six major pathways [24]; Second, the T6P pathway affects the expression of important flowering-related genes such as *SQUAMOSA PROMOTER BINDING PROTEIN LIKE* (*SPL*) via the age pathway [15]. Taken together, sugars may be involved in the regulation of flowering through multiple flowering pathways. To our knowledge, the role of sugars in twice flowering has not been studied, which prompted our current investigation on the interactions between sugars and twice flowering in *Magnolia*.

*Magnolia* is a traditional and important ornamental plant in urban greening, which has been cultivated for about 2000 years in China for its elegant flower shape and gorgeous flower color [25, 26]. Most species of *Magnolia* bloom once a year in spring, while a few can flower again in summer or early autumn [27]. The characteristic of twice flowering of *Magnolia* has greatly enhanced its ornamental and research value, and it is meaningful to understand the molecular mechanisms controlling secondary flowering in *Magnolia*. *Magnolia liliiflora* ‘Hongyuanbao’ is one of the twice flowering *Magnolia* varieties that bloom in both spring and summer [28]. In order to explore the factors affecting the secondary flowering, we investigated the biochemical metabolism during the flower buds differentiation in ‘Hongyuanbao’ and identified a variety of metabolic compounds that might influence the flowering process. Additionally, the expression levels of genes involved in the metabolic pathways were studied, which provided important clues to understanding the metabolic regulation of twice flowering and its underlying molecular mechanisms.

## Results

### Identification of two distinct periods of flower bud differentiations in M. liliiflora ‘Hongyuanbao’

**Fig. 1:**
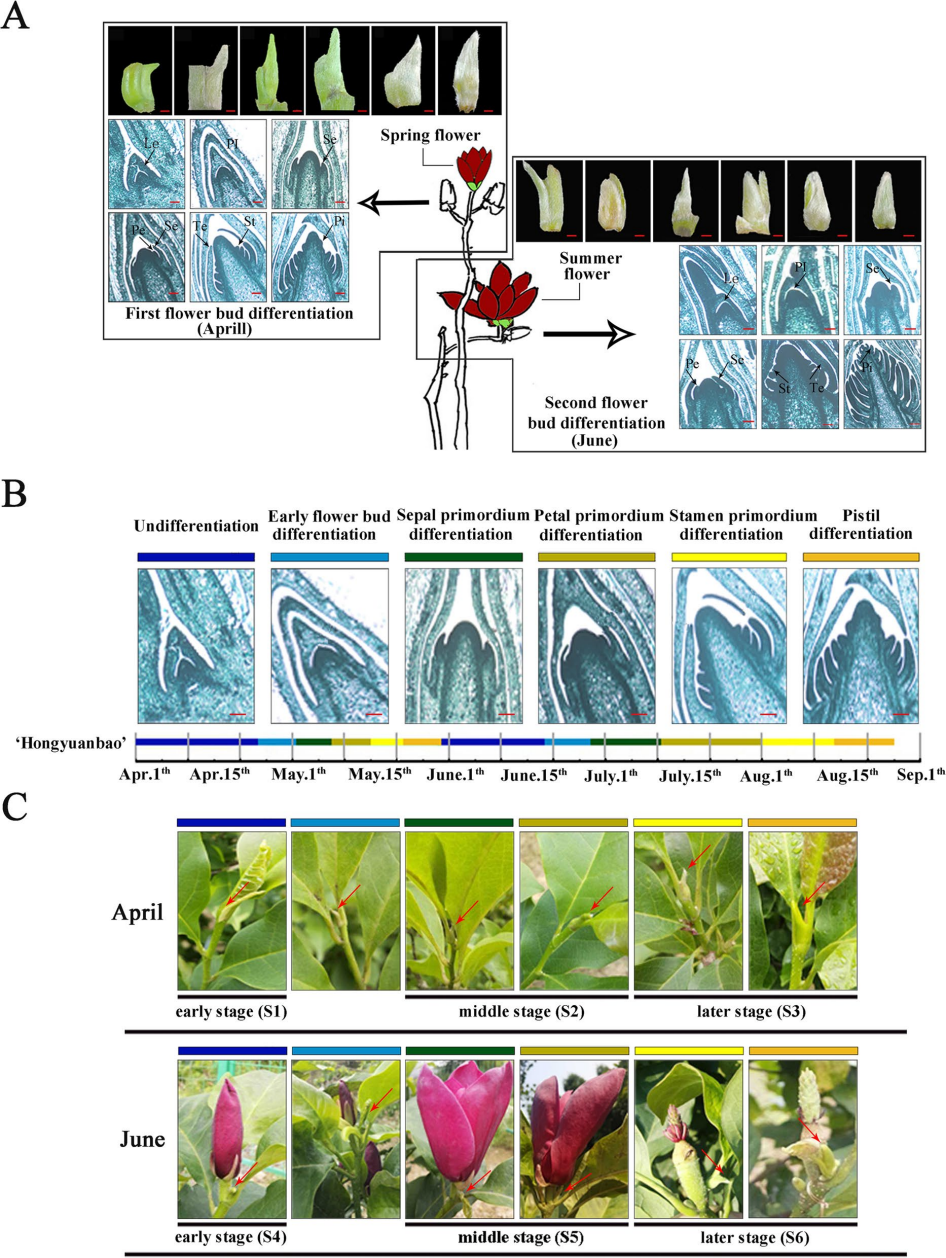
Observation of flower buds and plant phenotypes in *Magnolia liliiflora* ‘Hongyuanbao’. **A** The characteristic of twice flowering of ‘Hongyuanbao’. White bars, 2 mm; Red bars, 50 μm. **B** The time course of flower bud differentiation. Red bars, 50 μm. **C** The plant phenotypes changes of ‘Hongyuanbao’ during twice flowering process. S1, S2, S3 represent the early, middle, and later stage of the first flower bud differentiation, respectively. S4, S5, S6 represent the early, middle, and later stage of the second flower bud differentiation, respectively. The arrows pointed to the bud in April represent to the first flower bud differentiation process and the arrows in June represent to the second flower bud differentiation process

To reveal the flowering time and phenotypic changes of *M. liliiflora* ‘Hongyuanbao’, the entire flower bud differentiation process was observed by paraffin sections at regular intervals. Many *Magnolia* bloom once in either spring or summer, after completing the flower bud differentiation process during the previous year. Conversely, we found that ‘Houngyuanbao’ completed the differentiation process twice each year and flowered in both spring and summer (Fig. 1A). Based on the morphological features of floral bud differentiation in ‘Hongyuanbao’, we divided the process into the following six stages (also represented by different colors) (Fig. 1B): undifferentiated, early flower bud differentiation, sepal primordium differentiation, petal primordium differentiation, stamen primordium differentiation, and pistil primordium differentiation. Plant phenotype observation revealed that the spring flowers, which had completed flower bud differentiation in the previous year, were mainly produced at the top of the last year branches. As the falling of the spring flowers, new branches grown, together with the first time progress of bud differentiation on top the branches from April 22 to May 30. Then the first time flower buds turned to be bigger and bloomed on June (summer flower), as the second flower bud differentiation would begin and finished at the end of August. The flower bud of the second time would bloom in the next spring. (Fig. 1). This phenomenon appeared that variation existed in the development and opening process of flower buds between spring and summer, which possibly be attributed to variation in plant nutritional status and environmental conditions [9, 29].

### Untargeted metabolomic comparison of primary metabolites across the first and second flower bud differentiation

**Fig. 2:**
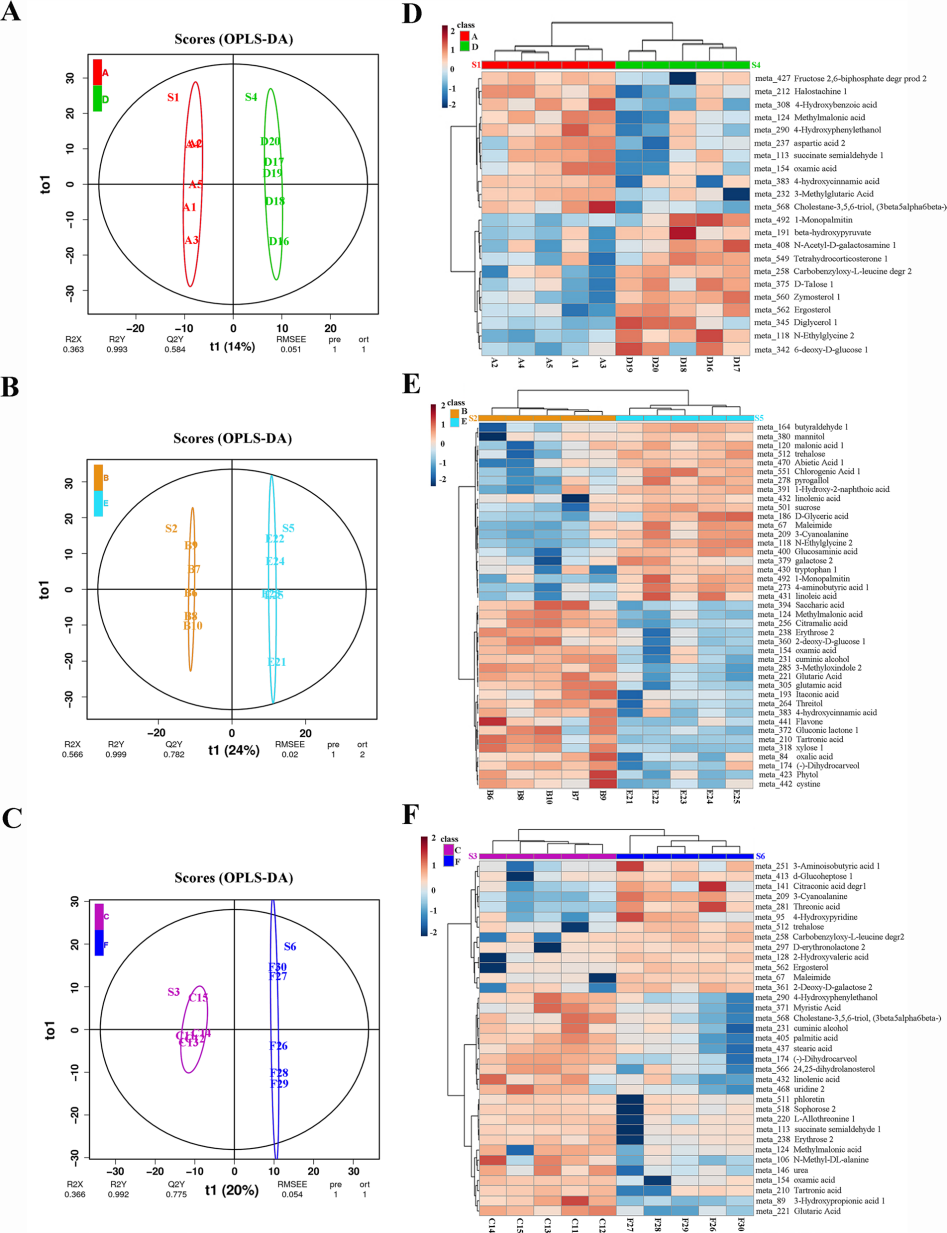
Comparisons of metabolite profiles in the first and second flower buds. **A**-**C** orthogonal projection to latent structures discriminant analysis (OPLS-DA) score plot for the different stages, comparing metabolite between the first and secondlower buds. *n* = 5. t1 represents the score values of the main components and reveals the differences between groups; to1 displays the score value of the orthogonal component and the difference within the group; Q2 represented the prediction ability of OPLS-DA model. **D-F** Heatmap of the classification in the different stage comparing between the first and second flower bud differentiations on metabolite profiles. S1, S2, S3 represent the early, middle, and later stage of the first flower bud differentiation, respectively. S4, S5, S6 represent the early, middle, and later stage of the second flower bud differentiation, respectively

To explore the mechanisms of the twice flowering trait, we harvested buds in April and June with early stage (undifferentiated, S1 & S4), middle stage (sepal primordium and petal primordium differentiation, S2 & S5), and late stage (stamen primordium and pistil primordium differentiation, S3 & S6) (Fig. 1). The primary metabolites of the two bud differentiation periods were analyzed using gas chromatography-mass spectrometry (GC-MS). In most metabolomic data analyses, confounding factors orthogonal to the variables of interest may obscure the intended class separation. To analyze these data, we compared two different analyses: principal components analysis (PCA) and orthogonal projection to latent structures discriminant analysis (OPLS-DA). For PCA, our classification variables was obscured, and the variables were dispersed across several principal components. Therefore, better visualization and subsequent analysis could not be carried out (Additional file 1). Thus, we tested OPLS-DA as an alternative method, which could filter out the dis-related orthogonal variables in classification variables. We were then able to analyze both the nonorthogonal and orthogonal variables (Fig. 2). The value of the Q2 is deemed to represent the prediction ability of OPLS-DA model, and for biological samples, Q2 ≥ 0.4 is ideal [30]. It showed that the OPLS-DA score plot for each comparison between the primary and secondary flower buds had overall cross-validation coefficients, and Q2(y) was 58, 78 and 77% respectively. It suggested that OPLS-DA model was more suitable than PCA for sample category prediction of ‘Hongyuanbao’. The coordinates value for to1 and t1 showed a clear separation of the first predicted component between the two groups (Fig. 2A-C), showing the inter-group and intra-group differences.

To investigate the relationship between metabolites and the twice flowering phenotype, the metabolic profiles of both the primary and the secondary flower buds were used to conduct hierarchical cluster analysis (Fig. 2). A total of 41 differential metabolites were identified (Additional file 2). Based on the Mass Bank and Kyoto Encyclopedia of Genes and Genomes (KEGG) and LECO-Fiehn Rtx5 Database analyses [31, 32], all 41 metabolites were analyzed and divided into six groups: Carboxylic acids and derivatives (including 12 compounds), Organooxygen compounds (13), Fatty Acyls (5), Prenol lipids (3), Indoles and derivatives (2), and other compounds (6). Compared to first flower bud differentiation, the second flower bud showed higher levels of most sugars and organic acids, while the levels of glutamate, pyroglutamate, malate, citrate and 2-oxoglutarate were decreased. It revealed that in second flower buds, sugars including D-Talose 1 and 6-deoxy-D-glucose 1 were increased at the early stage and the levels of sucrose, trehalose, galactose 2, xylose 1 and some other sugars at the middle stage were higher. The second flower buds had higher levels of certain sugars than first flower buds at the later stage, including trehalose, erythrose 2, and d-glucoheptose 1. The data suggested that many metabolites, especially sugars, are necessary for the differentiation of the second flower bud.

### Correlation among metabolites in M. liliiflora ‘Hongyuanbao’

**Fig. 3:**
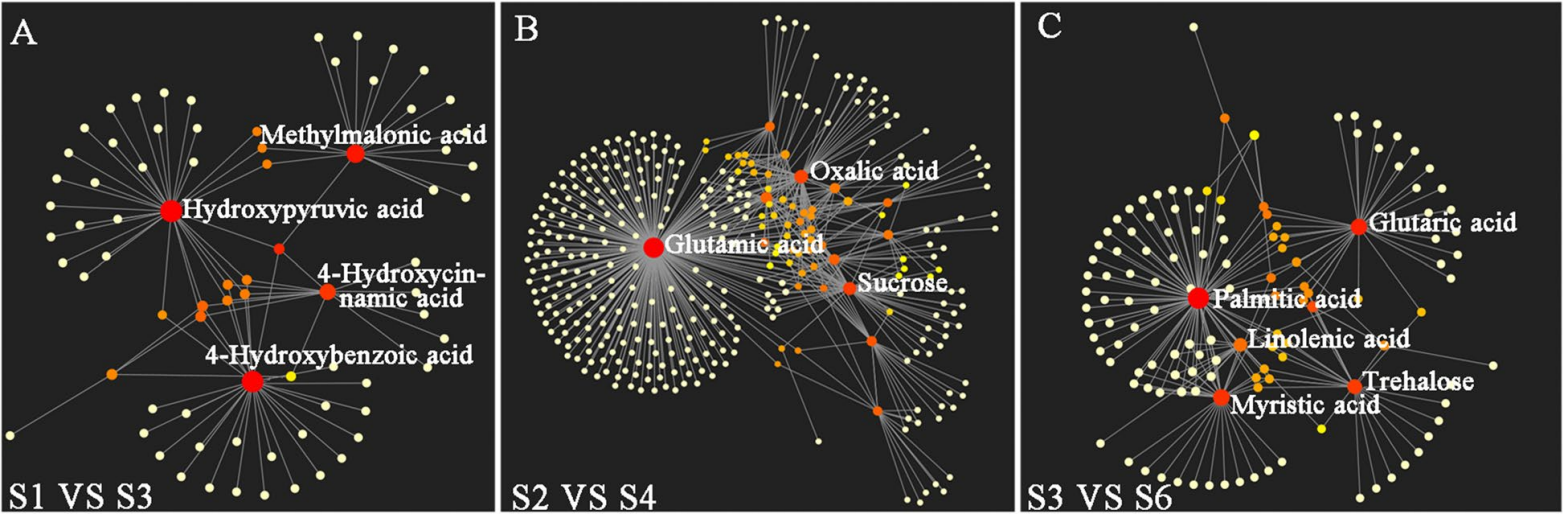
Metabolite-Metabolite Interaction Network in different stages. S1, S2, S3 represent the early, middle, and later stage of the first flower bud differentiation, respectively. S4, S5, S6 represent the early, middle, and later stage of the second flower bud differentiation, respectively. The circles of different sizes represent interactions between metabolites. The larger the circle, the more closely it interacts with other metabolites. The straight lines between the circles represent interactions between metabolites

To study the correlation between the metabolites in different flower bud differentiation stages of *M. liliiflora* ‘Hongyuanbao’, Pearson correlations were used to calculate the degree of similarity within metabolite profiles in the three different developmental stages. MetaboAnalyst 4.0 (http://www.metaboanalyst.ca) [33] was used to map the correlation network among metabolites. S1, S2, S3 and S4, S5, S6 represented the early (undifferentiation), middle (sepal differentiation and petal differentiation), and later (stamen differentiation and pistil differentiation) stages of the first and second bud differentiation respectively. It revealed that between the first and second flower buds, 22, 41, and 25 metabolites differed across the early, middle, and later stages (Fig. 3A-C). In order to screen key differential metabolites and metabolic pathways by analyzing the differential metabolite interactions at each flower bud differentiation stage, correlation networks were built. These networks showed that in the differential metabolites at the early stage, Methylmalonic acid, 4-hydroxycinnamic acid, 4-Hydroxybenzoic acid and Hydroxypyruvic acid interacted the most closely with other differential metabolites. Sucrose, Oxalic acid and Glutamic acid were found to interact the most closely with other differential metabolites at the middle stage. Glutaric acid, Palmitic acid, Myristic acid, Trehalose and Linolenic acid interacted the most closely with other differential metabolites at the later stage (Fig. 3). Besides that, it was found that the activity and association of metabolites were more prominent during the comparison of S2 and S5 than the comparison of S1 and S4, or S3 and S6 (Fig. 3). The results suggested that among those differential metabolites, the sugar metabolites were closely associated with other metabolites, and the sugar metabolism pathway might play a vital role during the first and second flower bud differentiation processes.

### KEGG enrichment analysis of sugar metabolites and the expression of MlTPS genes

**Fig. 4:**
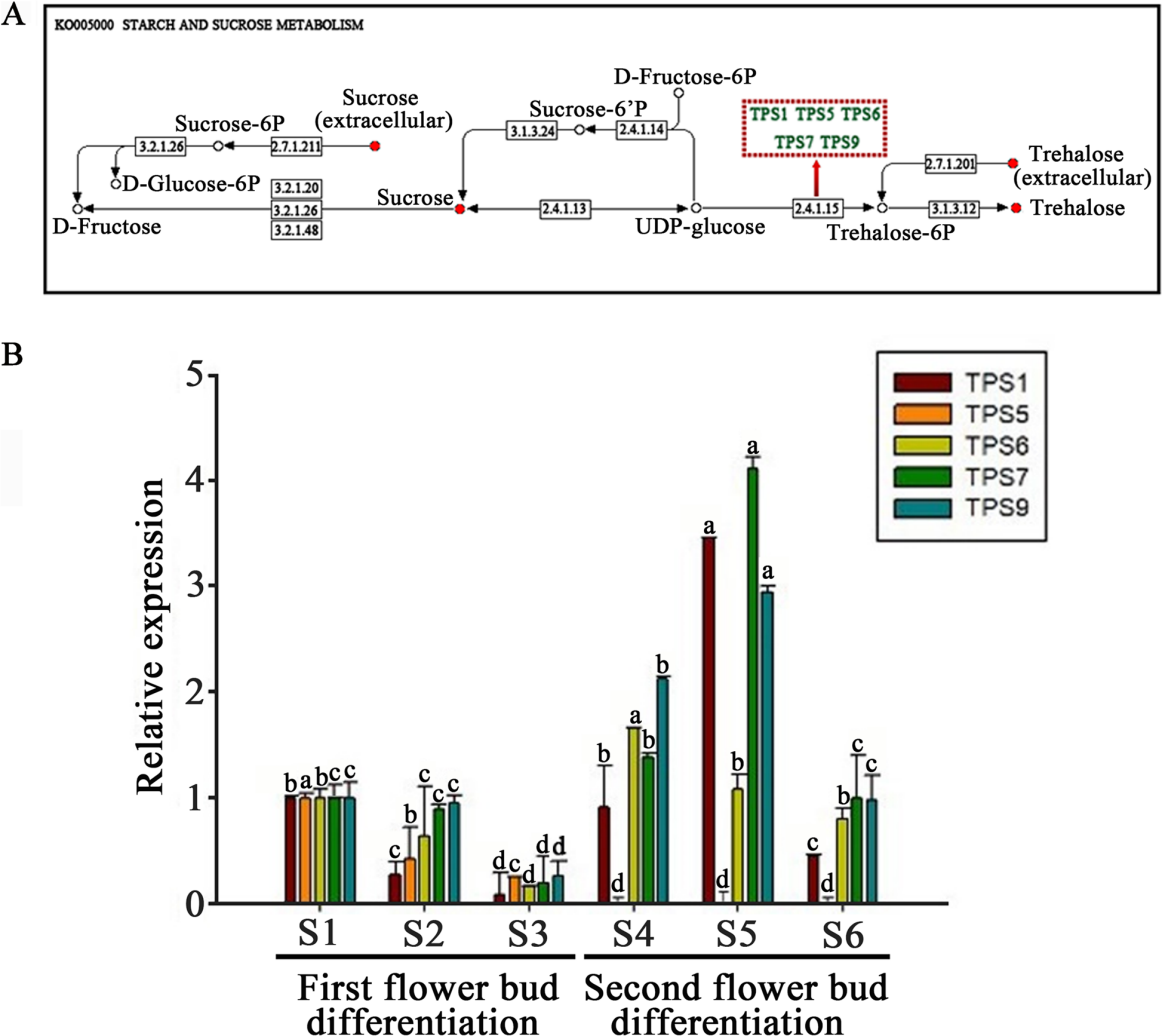
Important metabolite pathways during the first and second flower bud differentiations. **A** Enrichment of the Kyoto Encyclopedia of Genes and Genomes (KEGG) pathway. Relatively high numbers of metabolites were annotated as starch and sucrose metabolism with rich factor. **B** The levels of expression of *MlTPS* genes in the three stages of the first and second flower bud differentiation processes in *Magnolia liliiflora* ‘Hongyuanbao’. S1, S2, S3 and S4, S5, S6 represented the early (undifferentiation), middle (sepal differentiation and petal differentiation), and later (stamen differentiation and pistil differentiation) stage of the first and second flower bud differentiation, respectively. S1 was used as an internal control. (Mean ± SD, *n* = 3, *p* < 0.05)

The results of KEGG enrichment analysis showed that sucrose and trehalose in the sucrose and starch metabolic pathways were significantly up-regulated in the second flower buds than the first flower buds (Additional file 3, Fig. 4A). T6P is a signal of sucrose availability and is synthesized from UDP-glucose and glucose-6-phosphate by *TPS* genes [18, 21]. But only five *TPS* genes were found in transcriptome from the bud mixtures during twice flowering process, which named as *MlTPS1*, *MlTPS5*, *MlTPS6*, *MlTPS7* and *MlTPS9* respectively. The expression levels of *MlTPS* genes in twice flowering process were then analyzed by qRT-PCR. It showed that the expression levels of *MlTPS* genes had significantly differences during the twice differentiation process. Compared to the first flower bud differentiation, *MlTPS1*, *MlTPS7* and *MlTPS9* were the most prominent in expression, and *MlTPS5* was barely discernible in the second flower bud differentiation process (Fig. 4B). The overall trends demonstrated that the expression of *MlTPS* genes were increased significantly at the middle stage in the second flower bud differentiation (Fig. 4), suggestive of potential function role of *MlTPS* genes in the second flower bud differentiation.

### Trehalose promoted early flowering

**Fig. 5:**
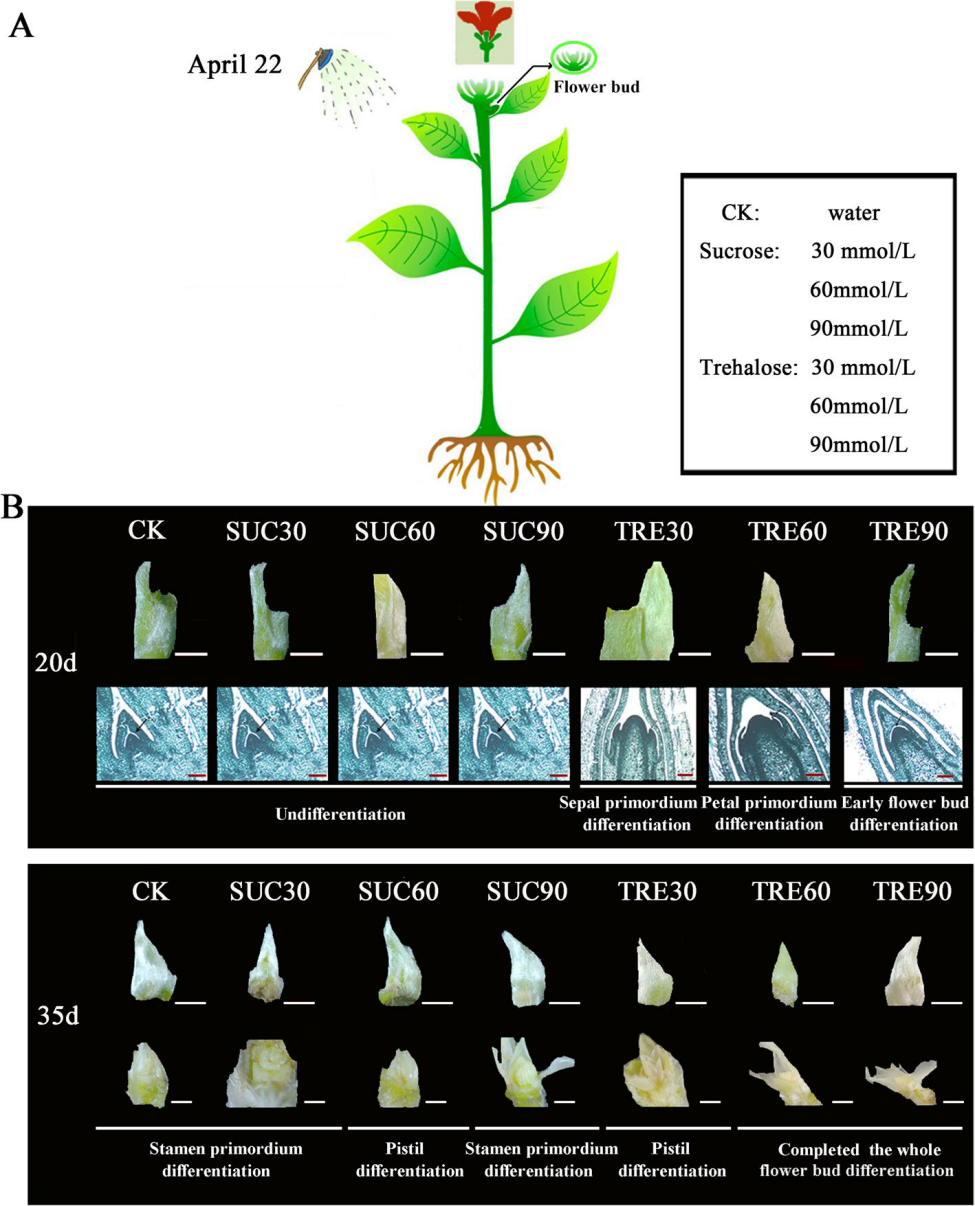
The effect of spraying on the leaves of *M. liliiflora* ‘Hongyuanbao’ plants with the solution of trehalose or sucrose. **A** Treatment with different concentrations of trehalose or sucrose solutions. The control and six treatment groups had three plants each. (*n* = 3). **B** The 20th and 35th day after treatment were chosen to observe the process of flower bud differentiation. Ten buds were harvested each time for dissection. Red bars, 50 μm; White bars, 2 mm

In order to confirm the effects of sucrose and trehalose on the twice flowering trait in *M. liliiflora* ‘Hongyuanbao’, the plants grown in the nursery of Zhejiang A&F University were sprayed with a solution of sucrose and trehalose once the plants finished the spring flowering on April 22, 2019. Leaf spraying commenced with three concentrations (30 mM, 60 mM, 90 mM) of sucrose and trehalose (Fig. 5A). The plants sprayed with deionized water were used as the control (CK). The leaves of all the plants were sprayed every 5 days and the samples were taken for microscopy observation on the 20th and 35th day following the first spraying.

The results of paraffin section showed that on the 20th day, plants treated with trehalose showed accelerated development relative to the control and sucrose treatment plants. Under trehalose treatments, buds had entered the floral transition, while the buds under sucrose treatments and the control remained undifferentiated (Fig. 5B). However, the rate of development did not correlate directly with trehalose concentration. Plants treated with 90 mmol/L trehalose had only reached early flower bud differentiation while plants treated with 30 and 60 mmol/L had reach sepal and petal primordium differentiation respectively. On the 35th day, both trehalose and sucrose treatments could promote floral development process. For plants treated with trehalose, the 60 and 90 mM treatments had completed flower bud differentiation, and the 30 mM had reached the pistil differentiation stage. The plants sprayed with 60 mM of sucrose were in the pistil developmental stage, while plants sprayed with 30 mM and 90 mM of sucrose were in stamen developmental stage, the same as CK. Taken together, these results demonstrate that spraying of ‘Hongyuanbao’ leaves with different concentrations of sugars, either sucrose or trehalose, have variable effects on flower bud differentiation, with trehalose showing prominent promotion effects on the process of flower bud differentiation (Fig. 5B).

### Expression of TPS genes and flowering-related genes under sucrose treatments

**Fig. 6:**
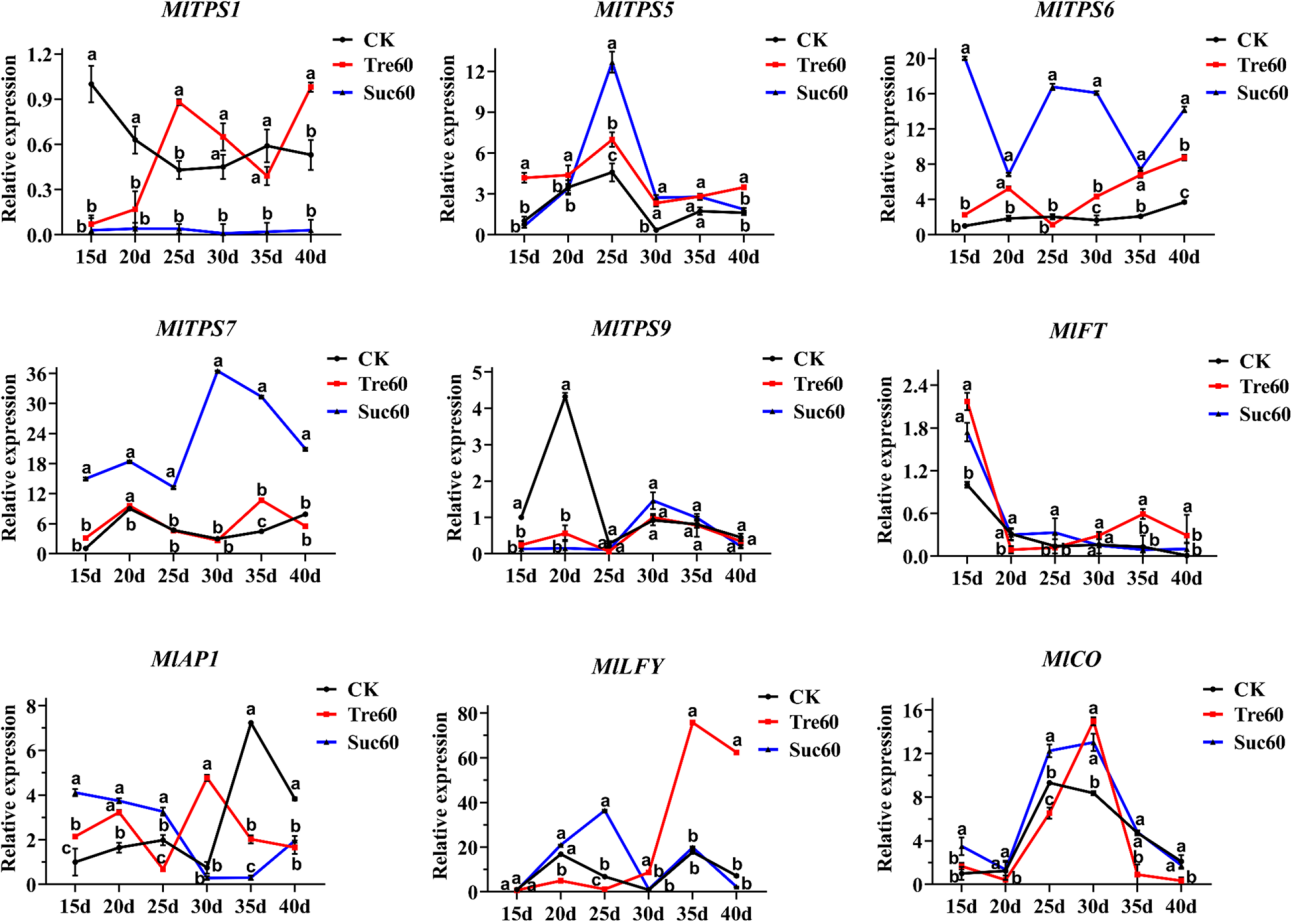
Expression levels of *TPS* and flowering genes in *Magnolia liliiflora* ‘Hongyuanbao’. 15d, 20d, 25d, 30d, 35d and 40d represented the days after the treatments with 60 mM of trehalose or sucrose. The bud’s stage at 20th day were undifferentiated under sucrose treatment and CK, and petal differentiation under trehalose treatment. At 35th day, the buds were stamen developmental stage under CK, pistil stage under sucrose treatment and accomplished the flower bud differentiation process trehalose treatment. (Mean ± SD, n = 3, *p* < 0.05)

To more clearly understand whether trehalose and sucrose influence the twice flowering phenotype, we explored the expression of both sugar-responsive genes and genes involved in flowering regulation. Specifically, we focused on the TPS family of *MlTPS1*, *MlTPS5*, *MlTPS6*, *MlTPS7* and *MlTPS9*, and key floral integrator genes *MlFT*, *MlLFY*, *MlCO*, and *MlAP1*. Previous studies have revealed that plants respectively sprayed with 60 mM of trehalose and sucrose could obviously promote flower bud differentiation process relative to the control and other treatment plants (Fig. 5B). Thus, the expression patterns of *MlTPS* genes and floral integrator genes in ‘Hongyuanbao’ leaves under treatments with 60 mM of trehalose and sucrose and CK condition were examined (Fig. 6).

It was apparent that the *MlTPS* genes were widely expressed throughout the flower bud differentiation period. In the treatment with a solution of 60 mM trehalose, relative to CK, *MlTPS1* and *MlTPS5* were increased during the middle (25d) and later stage (40d), and the expression of *MlTPS6* was decreased following an initial increase. While the expression of *MlTPS7* and *MlTPS9* remained unchanged. The results indicated that *MlTPS1* and *MlTPS5* might be response to trehalose treatment to regulate flowering. Under the treatment with 60 mM sucrose, the expression level of *MlTPS1* was barely detectable, and the expression of *MlTPS5* decreased following an initial increase. The expressions of *MlTPS6* and *MlTPS7* was always higher than that of the CK, and the *MlTPS9* expression remained unchanged, indicating that *MlTPS6* and *MlTPS7* might have an important function in sucrose treatment accelerating flowering. Moreover, the flowering integrators also responded to sugar treatment (Fig. 6). The expression of *MlFT*, which correlated with the timing of differentiation, was higher with trehalose and sucrose treatments than CK, suggesting that *MlFT* might play a vital role in flowering. The level of expression of *MlLFY* was higher in the beginning of flower differentiation compared to the later stages, suggestive of its functional role in the development of floral meristem. The expression of *MlCO* was raised moderately by the sugar treatments, while *MlAP1* was not responsive. These results demonstrated that *MlTPS* genes might be response to sugar signal to regulate floral differentiation and the acceleration in flowering promotion might depend on the enhanced expression of *MlFT* and *MlLFY*.

### Analysis of the expression of transcription factor SPL following sugar treatment

**Fig. 7:**
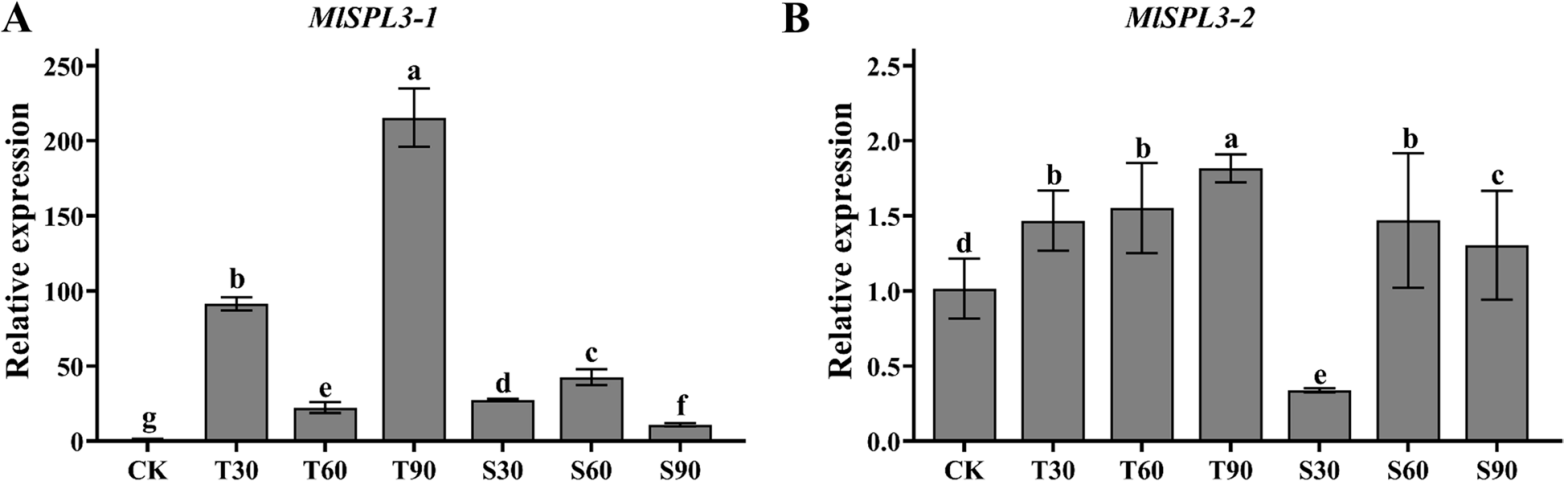
Analysis of the expression of *SPL3* gene after treatment with sugars. **A** The relative expression of *SPL3–1* on 20th day. (Mean ± SD, n = 3, *p* < 0.05). **B** The relative expression of *SPL3–2* on 20th day. (Mean ± SD, *n* = 3, *p* < 0.05)

*SPL* genes are regulated by diverse flowering signals and involved in age pathway to regulate flowering [34]. In other species, *SPL3* has been suggested as a participant in the T6P pathway and to affect the process of flowering [15]. Thus, we wanted to know whether *SPL3* homologous genes in ‘Hongyuanbao’ could respond to trehalose and sucrose treatments. We identified two *MlSPL3* genes (*MlSPL3–1* and *MlSPL3–2*) from the ‘Hongyuanbao’ transcriptome and determined whether they could respond to trehalose and sucrose treatments on 20th day. *MlSPL3–1* showed significantly higher expression relative to CK under both sugar treatments, with the trehalose treatments showing relatively higher expression than the sucrose treatment (Fig. 7A). While *MlSPL3–2* had significant expression under trehalose treatments, and the overall expression of *MlSPL3–2* was higher following spraying with trehalose than sucrose (Fig. 7B). These results suggest that the T6P pathway can affect the expression of flower-patterning gene *MlSPL3* via the age pathway to promote the floral differentiation process.

## Discussion

### Multiple mechanisms contribute to flowering in M. liliiflora ‘Hongyuanbao’

As the flowering time is critical for successful sexual reproduction, crop productivity and yield [34], numerous models have been established in *A. thaliana* and some crop plants. These models have also been used to study the molecular mechanisms that underlie the control of flower bud differentiation in ornamental plants. Nevertheless, the specific requirements for multiple flowering in ornamental plants have only recently become a popular research topic due to the increasing commercial interest [35, 36]. Flower bud differentiation is a physiological and morphological sign of the transformation from vegetative growth to reproductive growth [36]. The whole process consists of the induction stage before flower bud differentiation and the specific process of flower differentiation [37]. ‘Hongyuanbao’, a *M. liliiflora* variety, was introduced to China in 2001 [5]. Its ornamental features have been relatively stable for many years, especially the twice flowering phenotype. The phenomenon of twice flowering in ‘Hongyuanbao’ was found in one previous study [5], but the in-depth understanding on its flowering mechanism is still lacking. In this study, we made observations on the flowering time and the flower buds differentiation throughout the entire flowering period, and revealed that ‘Hongyuanbao’ had two distinct flower bud differentiations, the first and second differentiations in spring and summer respectively.

In recent years, increasing amounts of attention have been paid to plant metabolomics, and sugars have been deemed as signaling molecules that regulate a variety of genes involved in plant developments. In order to further uncover the potential mechanism underlying the twice flower bud differentiation in ‘Hongyuanbao’, the whole flower bud differentiation process was categorized into six stages. A metabolomic analysis of two different flowering buds across these six stages was performed and it suggested that the content of sugars increased significantly during the whole process in second flower bud differentiation. Sucrose and trehalose primarily occurred in the middle stage (sepal differentiation and petal differentiation) and trehalose also accumulated during the later stage (stamen differentiation and pistil differentiation). This indicated that sucrose and trehalose might play important roles during the process of flower bud differentiations, especially the secondary flower bud differentiation.

### Sucrose and trehalose play important roles during twice flower bud differentiation and blossom

Sugars, as the basic molecules of carbon metabolism, can be used as energy substances or signaling molecules in the entire life cycle of plants. A number of plant systems, such as Chrysanthemum [38], *A. thaliana* [15, 39], and apple [40, 41] have been well established for studying sugar metabolism. Recent studies found that sugars could influence the transition from vegetative growth to reproductive growth in plants, by acting as a signaling molecule which could interact with other inorganic regulatory networks [42, 43]. Consistent with previous research, metabolomics analysis of flower buds in ‘Hongyuanbao’ verifies the importance of sugars during flower bud differentiation process. In order to investigate the functional role of sugars during the twice flower bud differentiation in *M. liliiflora* ‘Hongyuanbao’, solutions of sucrose or trehalose were sprayed on plant leaves. It revealed that both sucrose and trehalose treatments could promote flower formation, with trehalose showing a more prominent effect than sucrose (Fig. 5).

**Fig. 8:**
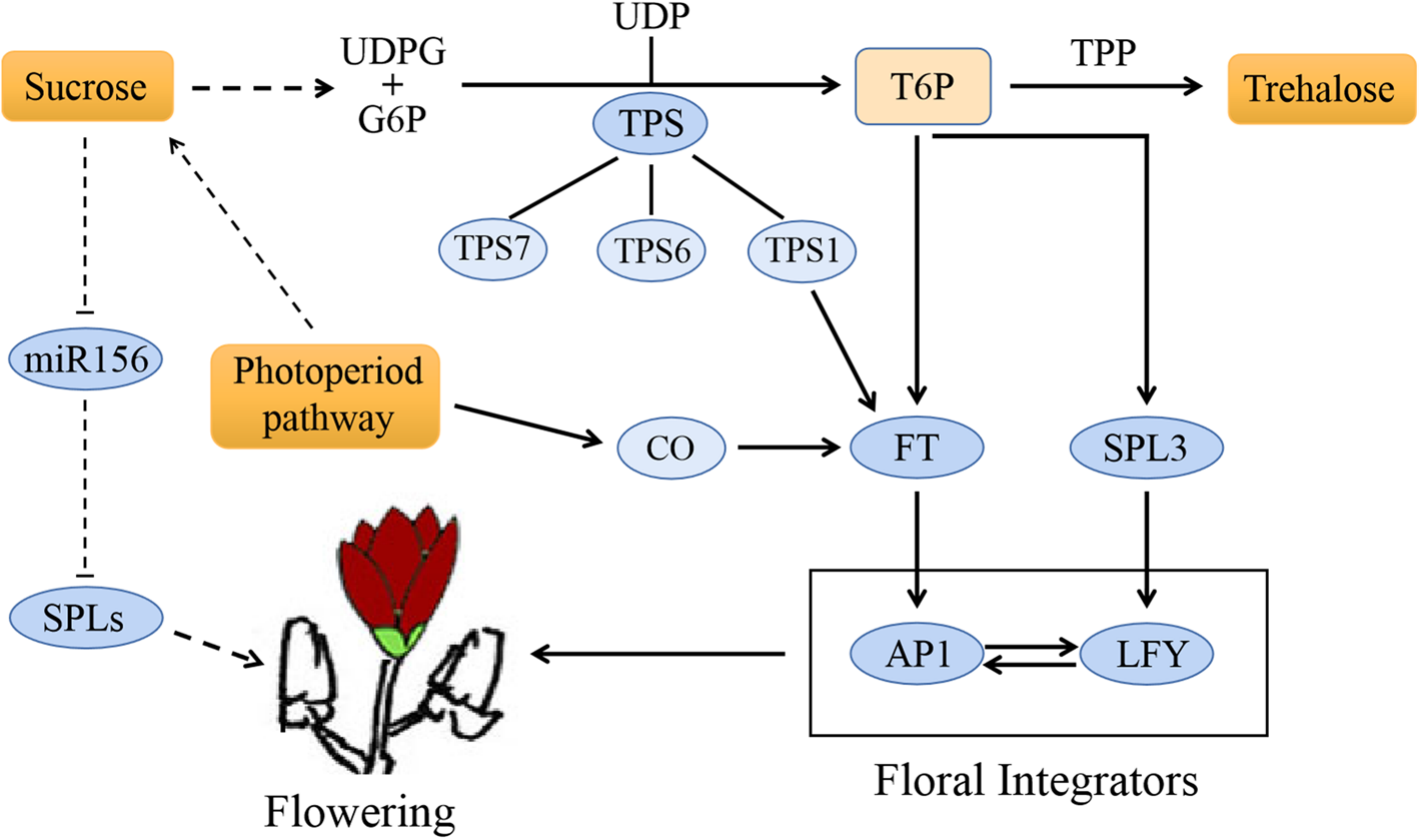
A simple schematic network of sugar metabolism that regulates flowering time in ‘Hongyuanbao’

As illustrated in Fig. 8, T6P acted as a signaling molecule that relayed information about carbohydrate availability to other signaling pathways, and played an important role in the sugar metabolism pathway [15]. The T6P pathway responds to sugar signaling and regulates flowering based on two different aspects. In the plant leaves, *TPS1* is activated by increasing daylength in spring, which produces T6P and induces the expression of florigen *FT* to regulate flowering [15, 42]. Besides that, the T6P pathway affects the expression of *SPL3* via the age pathway directly at the SAM independently of the daylength (Fig. 8).

Therefore, we screened and characterized five *MlTPS* and two *MlSPL3* genes from *M. liliiflora* ‘Hongyuanbao’. The expression of *MlTPS* genes in various stages were verified using qRT-PCR. It showed that *MlTPS1、MlTPS6、MlTPS7* were increased significantly under sugar treatment, indicating the possible function of *MlTPS* genes during flowering regulation. Previous studies in *A. thaliana* have also found that *AtTPS1* can promote flowering, while knocking out *AtTPS1* gene will result in delay in flowering [44]. However, the role of *AtTPS6* and *AtTPS7* in flowering regulation has not been studied further, and *AtTPS9* is involved in stress resistance [45]. Photoperiod is a daily recurring pattern of light and dark periods and can regulate sugar accumulation [46]. It is attempting to assume that the longer photoperiod during the timeframe of second flower bud differentiation leads to the accumulation of polysaccharides, which promote the expression of *MlTPS1* and the secondary flowering (Fig. 8). The T6P pathway may also function by the pathway of aging to promote flowering through the expression of *SPL3* [15]. In this study, we found that the activation of *MlSPL3* expression under treatments of sugars might induced by exogenous sugars treatments.

In conclusion, our study demonstrated that *M. liliiflora* ‘Hongyuanbao’ could go through two cycles of flower bud differentiations in spring and summer, distinct from the continuous flowering in roses and strawberry. Sucrose and trehalose are important players during the secondary flower bud differentiation and bloom likely by activating expression of *TPS* and *SPL* genes.

## Materials and methods

### Plant materials and growth conditions

The experiments were conducted with flower buds from three 10-year-old *M. liliiflora* ‘Hongyuanbao’, which were grown in the Zhejiang Agricultural and Forestry University, Zhejiang, China. All mature trees were managed according to ordinary culture practices. During the entire flower bud differentiation process, the flower buds were collected from the middle and upper regions of the trees crown every 5 days at about 2 pm from April 22 to August 21, 2018 for the follow-up observation and analysis. The buds for analysis were stored at − 80 °C until extraction.

### Morphological observation

To understand the flowering time and phenotypic changes of ‘Hongyuanbao’, a total of 10 flower buds from the three plants were collected every 5 days and stored in FAA fixative (8:1:1 ratio of 50% ethanol: formaldehyde: glacial acetic acid) for paraffin section observation. Samples were sliced to a 10-μm thickness and subjected to safranin fast green staining for 30 s. The samples were sealed with neutral balata and then observed and photographed using an Axio Imager A2 positive fluorescence microscope (Carl Zeiss, Oberkochen, Germany). At the same time, the development process of buds was observed using an anatomical microscope [47].

### Gas chromatography-mass spectrometry analysis

A total of 50 mg of each sample was placed in a 2 mL polyethylene tube, mixed with 400 μL cold extraction liquid (VMethanol: VH2O = 3:1), and 20 μL adonitol as the internal standard. A steel ball was added to the tube to assist the grinding with a 40 Hz grinder (JXFSTPRP-24, Shanghai Jingxin Experimental Technology, Shanghai, China). Following grinding for 4 min, the samples were treated with sonication in an ice bath for 5 min. The samples were then mixed with 200 μL of chloroform and 400 μL of water and centrifuged at 13,500x g for 15 min at 4 °C. Finally, 200 μL of supernatant was collected and evaporated by vacuum drying in a glass sampling vial. The samples were analyzed using gas chromatograph-mass spectrometry (GC-MS) Agilent 7890 GC-TOFMS which was equipped with an Agilent DB-5 ms capillary column (30 M × 250 μm × 0.25 μm, J & W Scientific, Folsom, CA, USA).

### Data processing and analysis

Chroma TOF 4.3X software of LECO Corporation and LECO-Fiehn Rtx5 database were used for raw peaks exacting, the data baselines filtering and calibration of the baseline, peak alignment, deconvolution analysis, peak identification and integration of the peak area. Both of mass spectrum match and retention index match were considered in metabolites identification. Remove peaks detected in < 50% of QC samples or RSD>30% in QC samples. SIMCA software (v.14) was used for multivariate pattern recognition and data normalization. Principal component analysis and the OPLS-DA method were used to discriminate the metabolic changes in the experimental group. The corresponding metabolic pathways were mapped in the KEGG database, and *P* values were calculated by Student’s T test with the level of statistical significance set at *p* < 0.05. To shrink any possible variance and to improve the performance for downstream statistical analysis, metabolite data were checked for data integrity and normalized using MetaboAnalyst4.0’s normalization protocols (selecting normalization by sum, log transformation, and auto-scaling) for statistical analysis. The heat map was generated by OriginPro2015 (OriginLab Corporation, Northampton, MA, USA).

### Screening and identification of metabolic differences

A total of 569 non-targeted metabolites were obtained based on the naming conditions of ion peak matching. All the differentially expressed compounds in the treated group were selected by comparing the compounds in the treated group with the control using the multivariate statistical method. For biologically duplicated metabolites, the combination of *P*-value and Variables with variable importance in the projection (VIP) value of the OPLS-DA (29 Thévenot E A, 2015) model was used to screen differential metabolites. The screening criteria were *P* < 0.05. VIP > 1.0 was considered relevant for group discrimination.

### RNA extraction, Illumina sequencing, assembly and annotation

Total RNA was extracted and enriched for poly(A) mRNA using the NEBNext Poly(A) mRNA Magnetic Isolation Module. Synthesis of cDNA for sequencing followed the strand-switching protocol from Oxford Nanopore Technologies. The cDNA library was sequenced on an Illumina HiSeq 4000 sequencing platform (Illumina, San Diego, CA, USA) by BIOMARKER TECHNOLOGIES (Beijing, China) to yield 2 × 150-bp paired-end raw reads. The sequenced raw reads were subjected to a quality check using FastQC [48]. The adapter sequences were removed from the raw reads. Reads with a ratio of ambiguous N nucleotides greater than 5% and those with low-quality sequences (quality score of less than 20) were removed. Sequencing reads were de novo assembled using Trinity software under default parameters and with a k-mer size of 25 [49]. The transcriptomes were assembled using pooled reads from all replications and stages. Assembly quality was critically assessed by BIOMARKER TECHNOLOGIES Company before subsequent analyses. The assembled transcriptome sequences were named ‘unigenes’. All unigenes were queried against six commonly used databases using BLASTx search to identify homologs (E-value < 10^− 10^). The databases used were Swiss-prot [50], Nr [4], KEGG [31], KOG [51], Pfam [52], and GO [53].

### The induction of flowering by sucrose and trehalose treatments

Twenty-one plants of similar size and growth were selected to grow in the Pingshan base experimental station. Once the plants finished the spring flowering, the leaves were sprayed from top to bottom with different sucrose or trehalose solutions with concentrations of 30 mM, 60 mM, and 90 mM on April 22, 2019. The plants sprayed with water were used as the control (CK). Each treatments, including CK, had three plants. The leaves of all the plants were sprayed once every 5 days and the samples were taken for microscopy observation and genes expression analysis on the 20th and 35th day following the first spraying. The stem tips were collected for cytological observation when the shoot apical meristems began to expand, which was used as the indicator of floral bud initiation.

### RNA extraction and the qRT-PCR analysis of MlTPS genes in response to sucrose treatment

Total RNA was extracted from ‘Hongyuanbao’ buds using the RNAprep Pure Plant Kit (TianGen, Beijing, China) and the quality was detected by nucleic acid analyzer (Implen Company in Germany). First-strand cDNA was synthesized by HiScript® III Reverse Transcriptase (Vazyme, Nanjing, China). The expression of genes including *MlTPS1*, *MlTPS5*, *MlTPS6*, *MlTPS7* and *MlTPS9*, and key floral integrator genes *MlFT*, *MlLFY*, *MlCO*, *MlAP1*, *MlSPL3–1* and *MlSPL3–2* with sequence similarity to the *Arabidopsis* genes were identified from the transcriptomic data of *M. liliiflora* ‘Hongyuanbao’ through the BlastX annotation, and detected by quantitative real-time polymerase chain reaction (qRT-PCR) on Light Cycller 480II Real Time PCR system (Roche, Basel, Switzerland). *MlACTIN* (*MlACT*) was used as a reference gene and its stability was analyzed in Additional file 4 [27]. Reaction system was: SYBR Premix ExTaq 10 μL, cDNA 2 μL, upstream and downstream primer (10 μmol/L) 8 μL each one, ddH_2_O supplemented to 20 μL. The reaction procedure was 95 °C for 30 s, 95 °C for 5 s, 60 °C for 30 s, a total of 40 cycles: 95 °C for 5 s, 60 °C for 1 min, 95 °C for 15 s. All qRT-PCR experiments were conducted with three biological replicates. The expression levels of genes between the control and treatment groups were compared. Statistical significance was calculated by Anova analysis followed by TukeyHSD. The genes expression pattern were performed using SigmaPlot 10.0 (Systat Software Inc., San Jose, CA, USA), and the relative transcript abundances were calculated using the 2^−∆∆Ct^ method [22].

## Supplementary Information

**Additional file 1: Fig. S1.** Comparisons of metabolite levels in spring and summer flower bud differentiations. Group A, B, C represent the early, middle, and later stage of the first flower bud differentiation, respectively. Group D, E, F represent the early, middle, and later stage of the second flower bud differentiation, respectively.

**Additional file 2. **All 41 metabolites occurring during various flower buds differentiation stages.

**Additional file 3: Fig. S1.** Scatter plot of Kegg Enrichment Results at the early stage between the first and second flower bud differentiation process. Enrichment factor of Oxidative phosphorylation was 14.83571. **Fig. S2.** Scatter plot of Kegg Enrichment Results at the middle stage between the first and second flower bud differentiation process. Enrichment factor of Starch and sucrose metabolism was 1.871292. **Fig. S3.** Scatter plot of Kegg Enrichment Results at the later stage between the first and second flower bud differentiation process. Enrichment factor of Starch and sucrose metabolism was 5.214849. **Fig. S4.** A standard Kegg output of starch and sucrose metabolism at the middle stage between the first and second flower bud differentiation process.

**Additional file 4: Table 1.** Primers for genes quantitative real-time PCR (q-PCR). **Table 2.** The CT values of *Actin* across the treatments. **Figure 1. **Expression level of *MlACT Magnolia liliiflora* ‘Hongyuanbao’. 15d, 20d, 25d, 30d, 35d and 40d represented the days after the treatments with 60 mM of trehalose or sucrose.

**Additional file 5: Table 1.** Differential metabolites between the first and second flower bud differentiations in three different developmental stages.

**Additional file 6: Table 1.** All metabolite profiles.

## Data Availability

All data generated or analyzed during this study are included in this published article and the additional files. Any additional data generated during the current study are available from the corresponding author on reasonable request.

## Abbreviations

KEGG: Kyoto Encyclopedia of Genes and Genomes
TPS: Trehalose-6-phosphate synthase
T6P: Trehalose-6-phosphate
OPLS-DA: Orthogonal projection to latent structures discriminant analysis
CK: Plants without any treatment
FT: FLOWERING LOCUS T
LFY: LEAFY
AP1: APETALA1
