# Supplementary material for: Metabolic analysis of the regulatory mechanism of sugars on secondary flowering in Magnolia
Source: BMC Mol Cell Biol. 2022 Dec 14;23:56. doi: 10.1186/s12860-022-00458-x (PMC9753265; doi:10.1186/s12860-022-00458-x)
Supplement: Supplementary file 1 — Additional file 1: Fig. S1. Comparisons of metabolite levels in spring and summer flower bud differentiations. Group A, B, C represent the early, middle, and later stage of the first flower bud differentiation, respectively. Group D, E, F represent the early, middle, and later stage of the second flower bud differentiation, respectively. [file 12860_2022_458_MOESM1_ESM.docx]

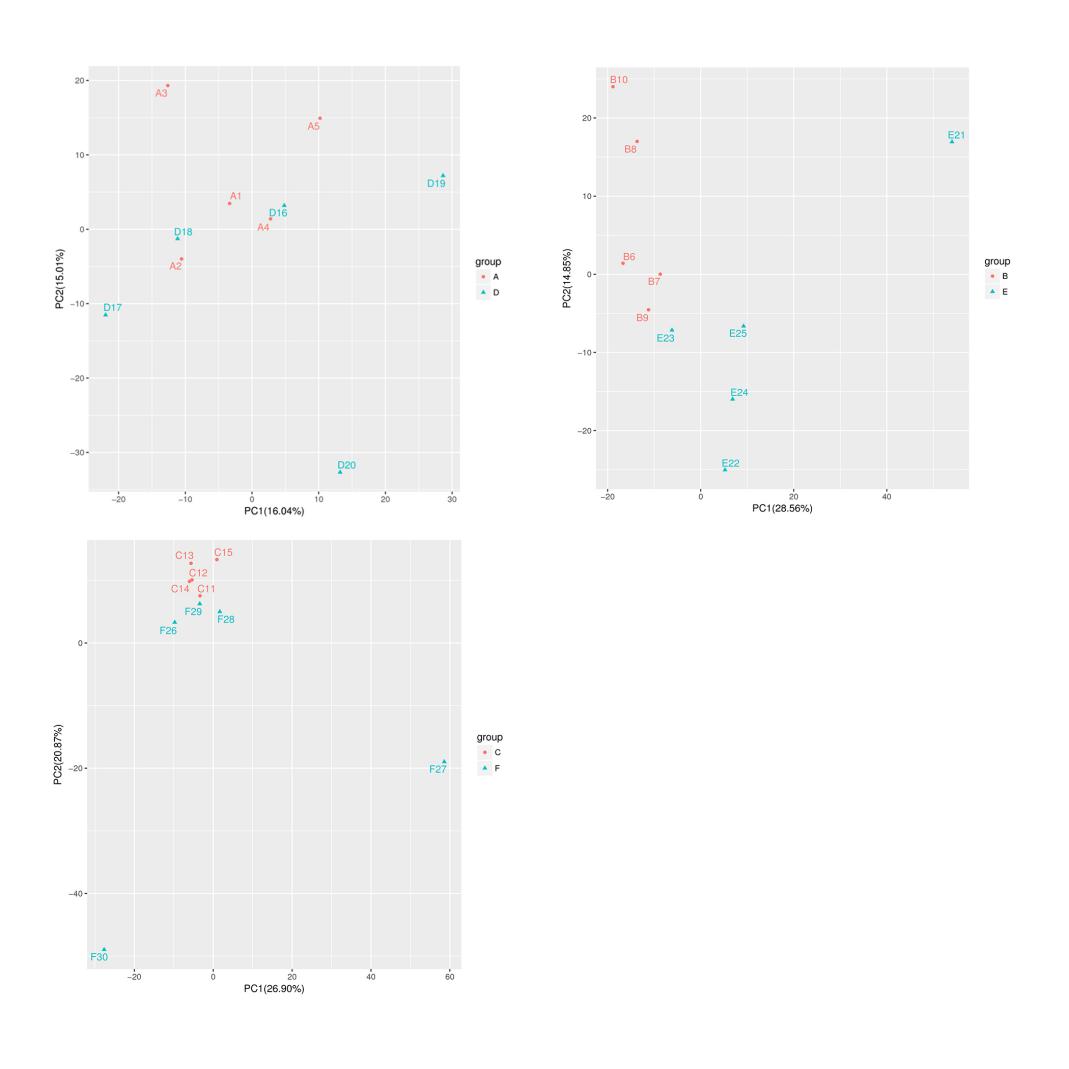


**Supplemental Figure S1.** Comparisons of metabolite levels in spring and summer flower bud differentiations.

Group A, B, C represent the early, middle, and later stage of the first flower bud differentiation, respectively. Group D, E, F represent the early, middle, and later stage of the second flower bud differentiation, respectively.
