# Supplementary material for: Metabolic analysis of the regulatory mechanism of sugars on secondary flowering in Magnolia
Source: BMC Mol Cell Biol. 2022 Dec 14;23:56. doi: 10.1186/s12860-022-00458-x (PMC9753265; doi:10.1186/s12860-022-00458-x)
Supplement: Supplementary file 3 — Additional file 3: Fig. S1. Scatter plot of Kegg Enrichment Results at the early stage between the first and second flower bud differentiation process. Enrichment factor of Oxidative phosphorylation was 14.83571. Fig. S2. Scatter plot of Kegg Enrichment Results at the middle stage between the first and second flower bud differentiation process. Enrichment factor of Starch and sucrose metabolism was 1.871292. Fig. S3. Scatter plot of Kegg Enrichment Results at the later stage between the first and second flower bud differentiation process. Enrichment factor of Starch and sucrose metabolism was 5.214849. Fig. S4. A standard Kegg output of starch and sucrose metabolism at the middle stage between the first and second flower bud differentiation process. [file 12860_2022_458_MOESM3_ESM.docx]

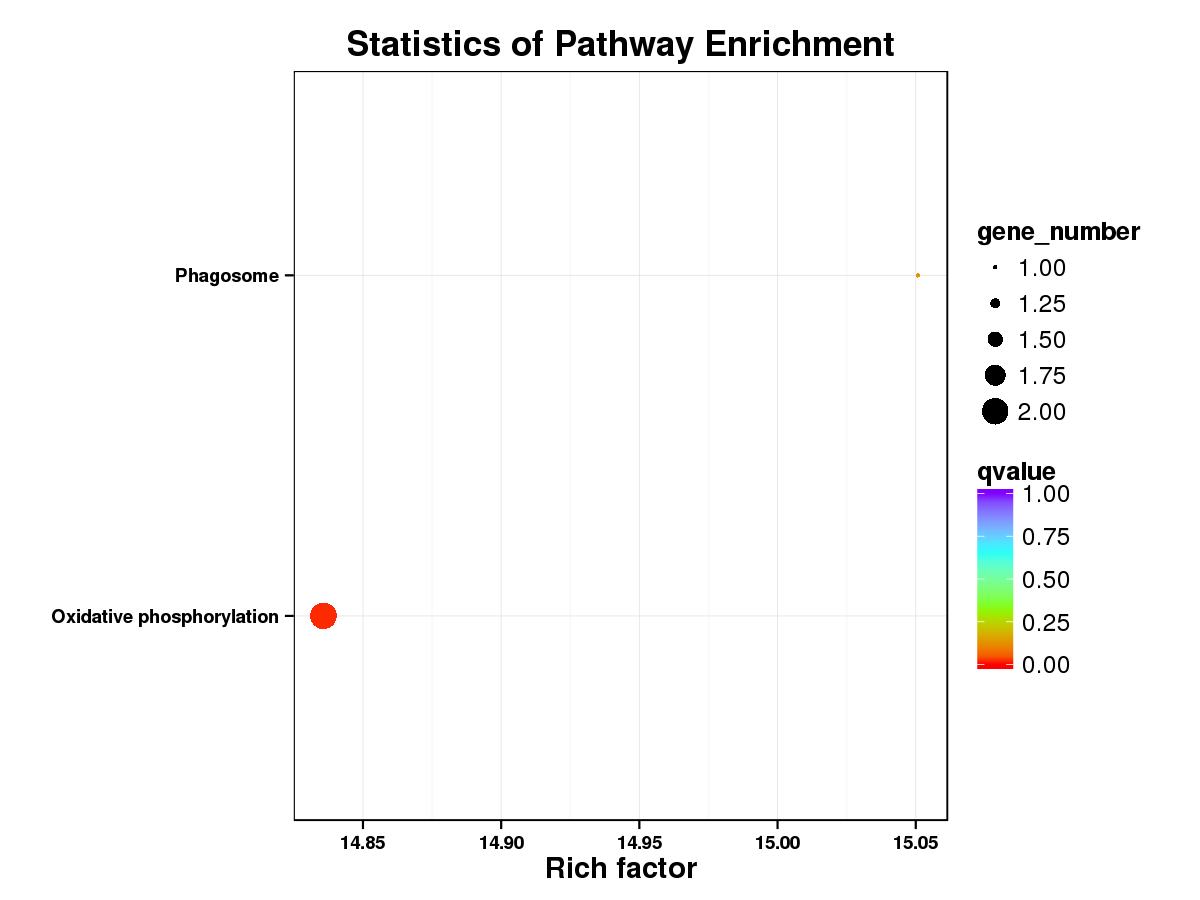


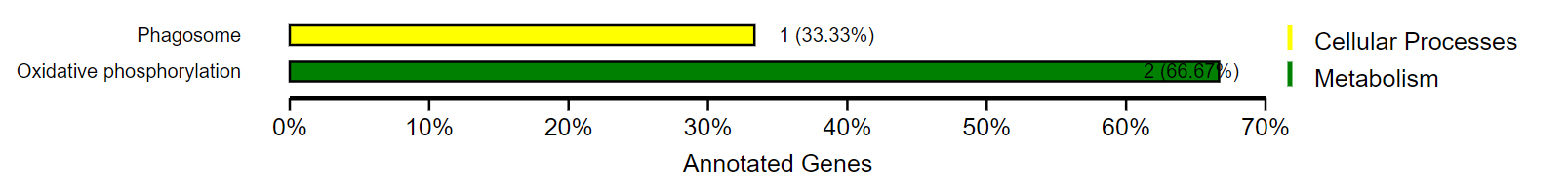


Supplemental Figure S1. Scatter plot of Kegg Enrichment Results at the early stage between the first and second flower bud differentiation process. Enrichment factor of Oxidative phosphorylation was 14.83571


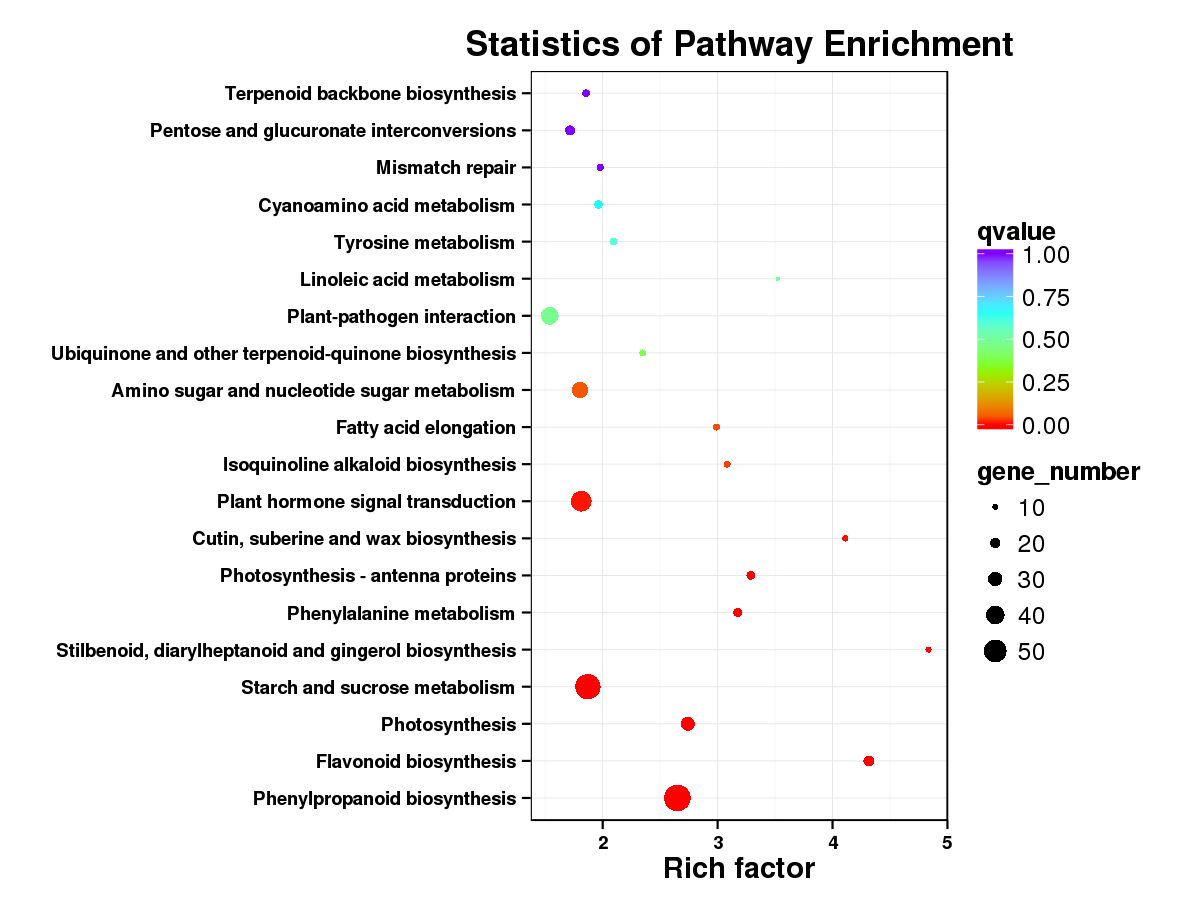


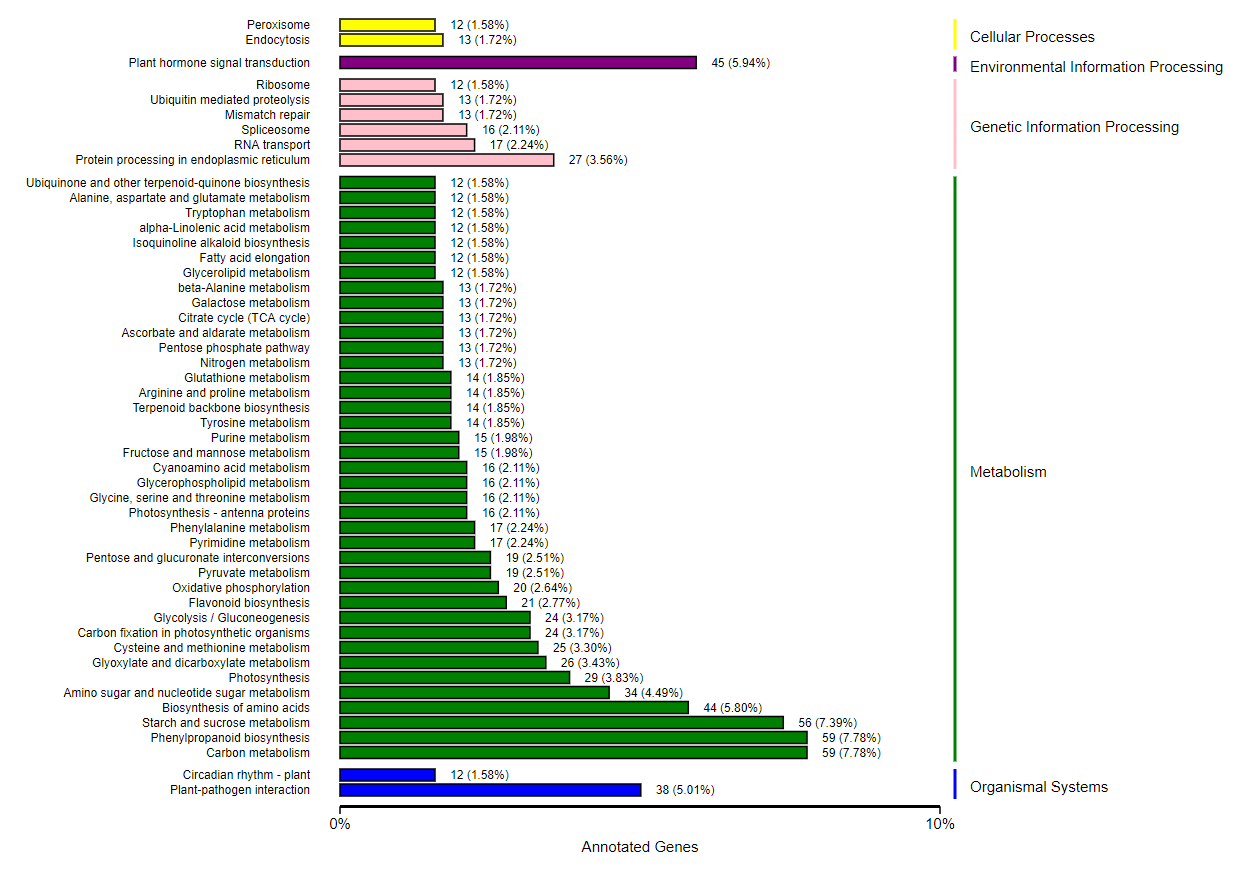


Supplemental Figure S2. Scatter plot of Kegg Enrichment Results at the middle stage between the first and second flower bud differentiation process. Enrichment factor of Starch and sucrose metabolism was 1.871292


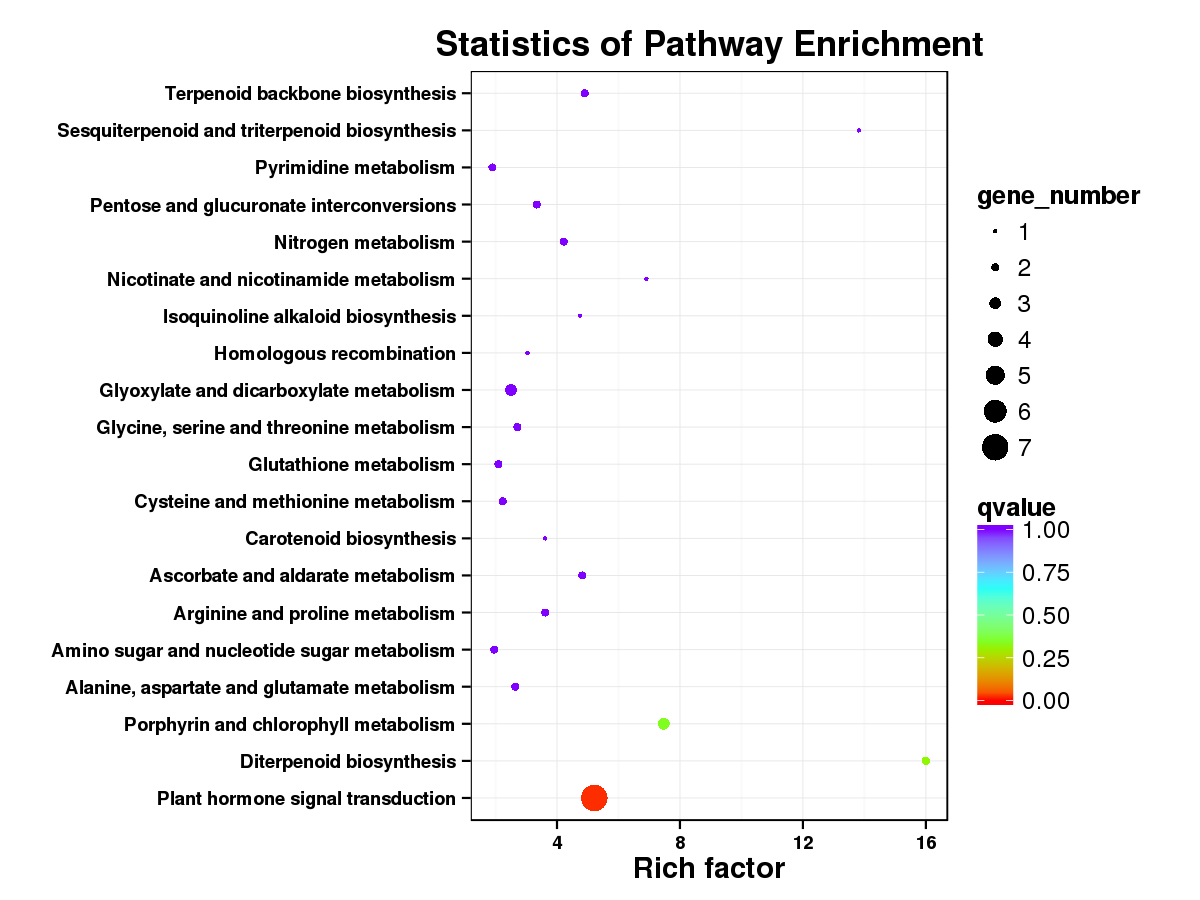


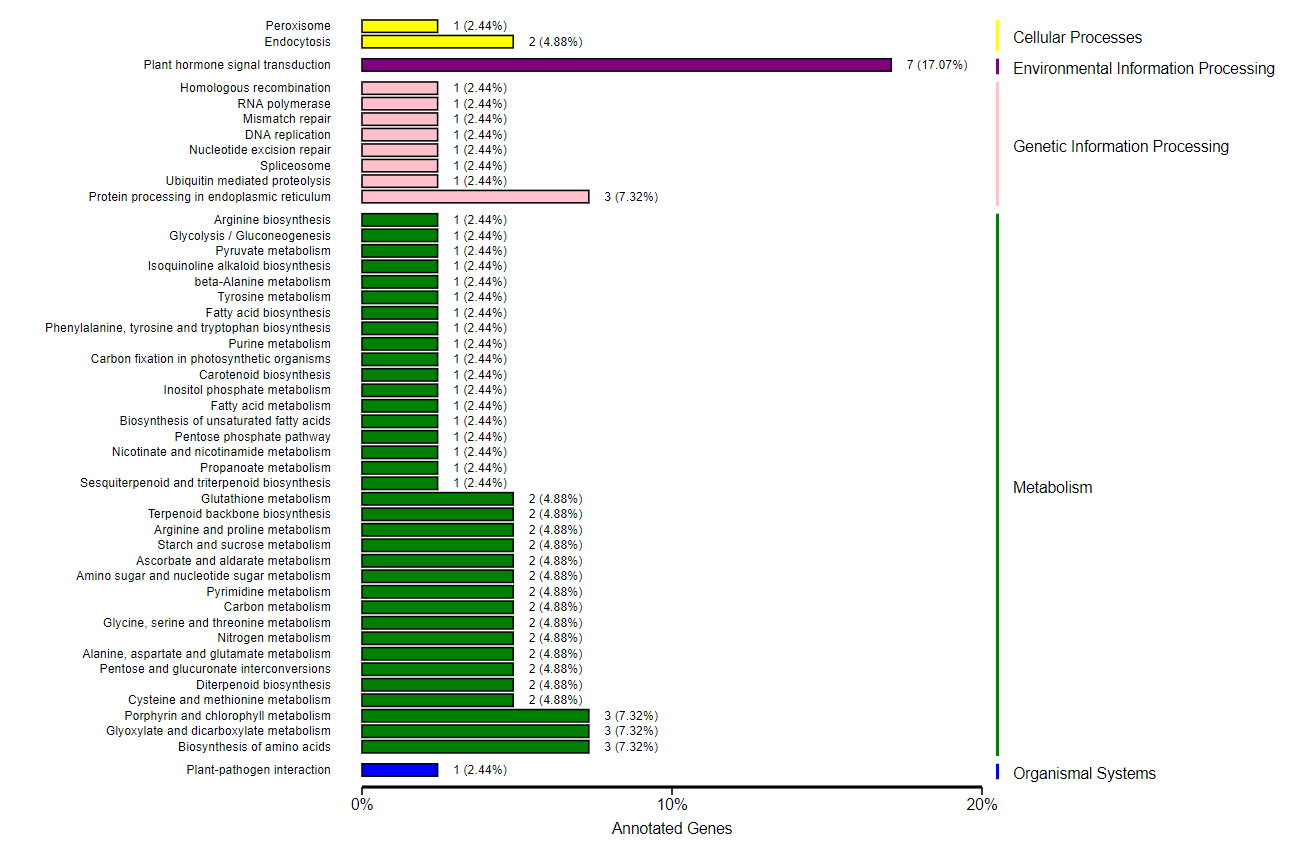


Supplemental Figure S3. Scatter plot of Kegg Enrichment Results at the later stage between the first and second flower bud differentiation process. Enrichment factor of Starch and sucrose metabolism was 5.214849


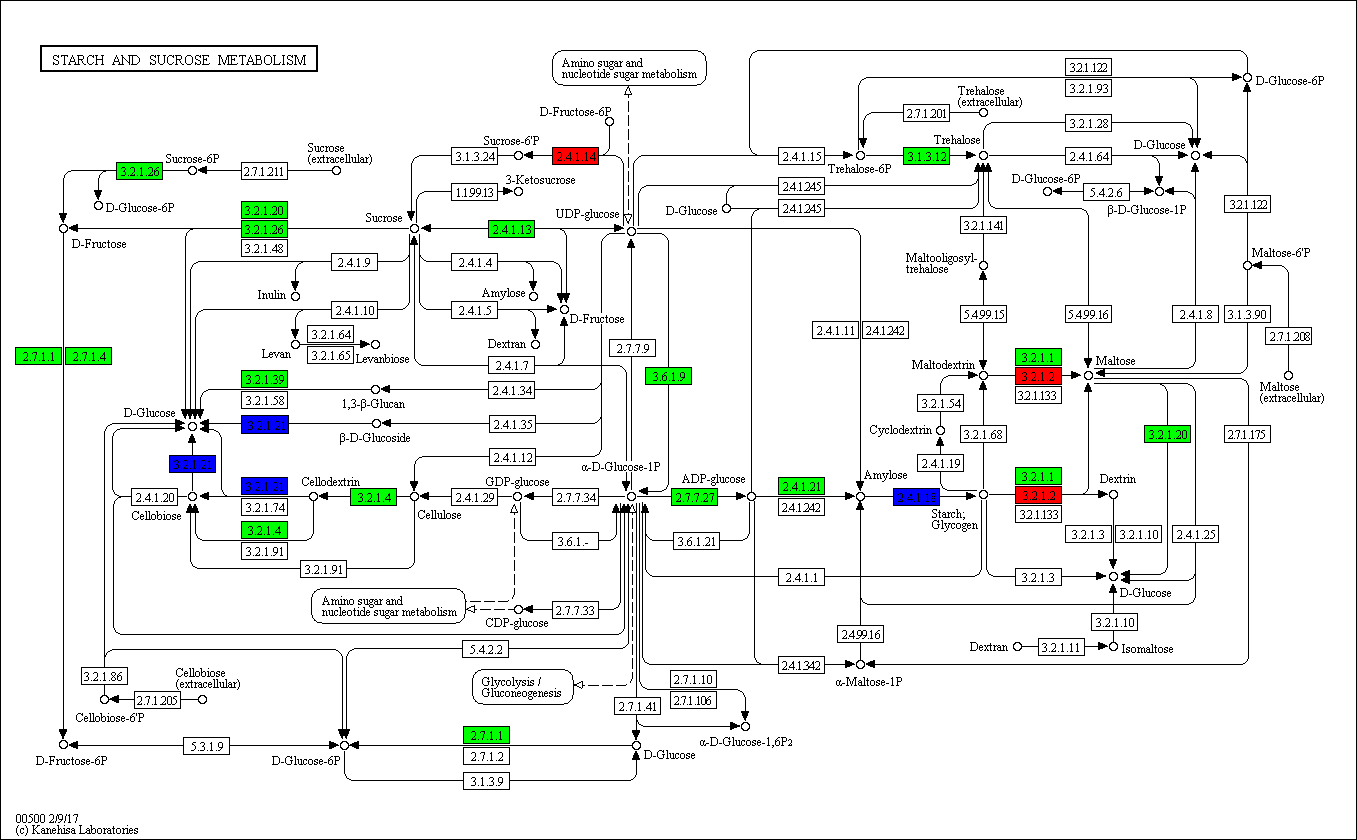


Supplemental Figure S4. a standard Kegg output of starch and sucrose metabolism at the middle stage between the first and second flower bud differentiation process.
