# Supplementary material for: Metabolic analysis of the regulatory mechanism of sugars on secondary flowering in Magnolia
Source: BMC Mol Cell Biol. 2022 Dec 14;23:56. doi: 10.1186/s12860-022-00458-x (PMC9753265; doi:10.1186/s12860-022-00458-x)
Supplement: Supplementary file 4 — Additional file 4: Table 1. Primers for genes quantitative real-time PCR (q-PCR). Table 2. The CT values of Actin across the treatments. Figure 1. Expression level of MlACT Magnolia liliiflora ‘Hongyuanbao’. 15d, 20d, 25d, 30d, 35d and 40d represented the days after the treatments with 60 mM of trehalose or sucrose. [file 12860_2022_458_MOESM4_ESM.docx]

Table 1 Primers for genes quantitative real-time PCR (q-PCR)

| （Gene） | （Primer name） | （Primer sequence(5' - 3')） |
| --- | --- | --- |
| *TPS1* | TPS1-F | ATGGGGATGTTGTTTGG |
|  | TPS1-R | TATCCCGATAGGAAACG |
| *TPS5* | TPS5-F | CTTAGGGACCAGTTTCA |
|  | TPS5-R | CTTCCCTAACCAGTCGG |
| *TPS6* | TPS6-F | GTATGCTTGGGCTCACC |
|  | TPS6-R | AAGGCTTAGGACTGACT |
| *TPS7* | TPS7-F | ACACCATTGTCTCGGCATA |
|  | TPS7-R | CGGGGTTTTGTTGATAGAG |
| *TPS9* | TPS9-F | TTATGCCCGCCATTTCC |
|  | TPS9-R | GCCCATAATACTCCAATCCAAT |
| *FT* | FT-F | CTCCAGGTTGGCGGCAGAAT |
|  | FT-R | TACACTGCGGCAACC |
| *LFY* | LFY-F | GCGTCCGCTACTACA |
|  | LFY-R | CGTCGATCTCCAAGTC |
| *CO* | CO-F | GGCGGTGATTCCAGCAT |
|  | CO-R | CGTGATGCGACAGGGTT |
| SPL3-1 | SPL3-1-F | CGACGACTGTGATGC |
|  | SPL3-1-R | TGACCTGCCAACCGT |
| SPL3-2 | SPL3-2-F | CGACTGTGATGCTGAC |
|  | SPL3-2-R | TGTGACCTGCCAACC |
| *Actin* | Actin-F | ACGAATCCGGTCCATCCATT |
|  | Actin-R | CCGTTCCACCAGGCAATATG |

Table 2 The CT values of *Actin* across the treatments

|  | 15d | | | 20d | | | 25d | | | 30d | | | 35d | | | 40d | | |
| --- | --- | --- | --- | --- | --- | --- | --- | --- | --- | --- | --- | --- | --- | --- | --- | --- | --- | --- |
| *Actin* | CK | Tre60 | Suc60 | CK | Tre60 | Suc60 | CK | Tre60 | Suc60 | CK | Tre60 | Suc60 | CK | Tre60 | Suc60 | CK | Tre60 | Suc60 |
| Sample-1 | 18.69 | 18.89 | 18.42 | 19.36 | 18.98 | 19.11 | 19.33 | 18.39 | 19.39 | 19.25 | 18.54 | 18.69 | 19.23 | 19.31 | 18.93 | 19.43 | 20.50 | 19.33 |
| Sample-2 | 18.74 | 18.77 | 18.47 | 19.45 | 19.29 | 18.94 | 19.32 | 18.34 | 19.36 | 19.3 | 18.59 | 18.74 | 19.24 | 19.16 | 18.98 | 19.36 | 20.96 | 19.32 |
| Sample-3 | 18.82 | 18.85 | 18.63 | 19.38 | 18.99 | 18.79 | 19.19 | 18.41 | 18.99 | 19.34 | 18.85 | 18.82 | 19.52 | 19.62 | 18.77 | 19.27 | 20.85 | 19.19 |


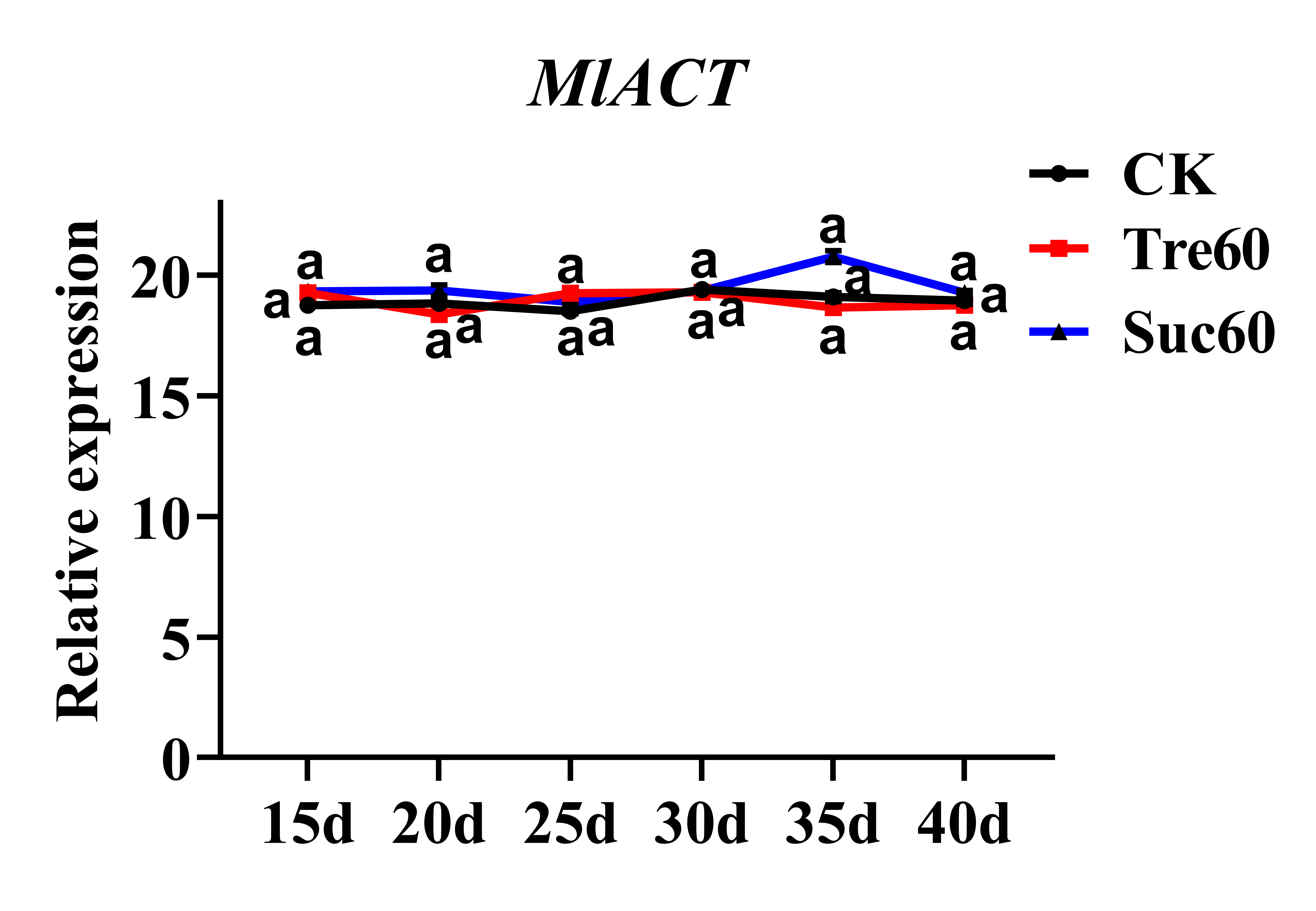


**Figure 1.** Expression level of *MlACT* *Magnolia liliiflora* ‘Hongyuanbao’. 15d, 20d, 25d, 30d, 35d and 40d represented the days after the treatments with 60 mM of trehalose or sucrose.
